# Risk of mortality from suicide in patients with Huntington’s disease is increased compared to the general population in England

**DOI:** 10.1007/s00415-022-11085-z

**Published:** 2022-03-27

**Authors:** Danah Alothman, Charles R. Marshall, Edward Tyrrell, Sarah Lewis, Timothy Card, Andrew Fogarty

**Affiliations:** 1 School of Medicine, University of Nottingham, Nottingham, UK; 2 Preventive Neurology Unit, Wolfson Institute of Population Health, London, EC1M 6BQ UK

**Keywords:** Huntington’s disease, Suicide, Neurodegenerative disease, Neuropsychiatry, Suicide risk

## Abstract

**Objective:**

To examine the relative risk of suicide mortality in patients with Huntington’s disease (HD).

**Methods:**

A case–control study design was used. We used linked electronic records from primary care, secondary care and Office for National Statistics from England from 2001 through 2019. Controls were matched to cases by general practice and suicide date. Odds ratios (ORs) were adjusted for gender and age at suicide/index date.

**Results:**

Data were available for 594,674 individuals. Patients with HD who died from suicide were significantly younger at time of death than patients with HD who died from causes other than suicide (*p* < 0.001). The adjusted OR for HD was 9.2 (95% confidence intervals, CI 4.9–17.4) compared to those without HD. The increase in risk was higher amongst the younger age group who were ≤ 45.8 years at suicide/index date (OR 54.5, 95% CI 10.8–276.1).

**Conclusion:**

The markedly elevated suicide risk in patients with HD suggests that implementation of suicide risk assessment may improve survival in individuals with these diseases, especially in younger patients.

**Supplementary Information:**

The online version contains supplementary material available at 10.1007/s00415-022-11085-z.

## Introduction

Huntington’s disease (HD) is an autosomal dominant inherited neurodegenerative condition characterized by motor, psychiatric and cognitive disturbances that generally manifests in middle-aged patients and has a median survival of 24 years from diagnosis [1]. HD can cause substantial disability and eventually leads to death. Amongst the top causes of death in patients with HD is suicide, immediately after pneumonia and infectious diseases [1].

Elevated rates of suicide in patients with HD were demonstrated in case series studies [2–4] with rates ranging between 5.7% [3] and 12.7% [4]. These results were considerably higher than the suicide rate in the general population (13.7 per 100 000) [5] suggesting an increased risk in patients with HD. Erlangsen et al. quantified the risk of suicide using hospital records for patients with HD [6]. Although the authors reported an increased risk with an adjusted rate ratio of 9.5, this patient population probably represents individuals with relatively advanced disease making the generalisability of these results to the general population challenging. We used data from population-based primary care databases to explore the relative risk of patients with HD dying from suicide compared to the general population in England. An improved understanding of suicide risk in patients with HD is important to inform suicide risk assessment for health care professionals potentially preventing premature death in those patients.

## Methods

### Study design and participants

The study design was a population-based case–control study in England from 2001 through 2019 using multiple integrated electronic records. We employed the Clinical Practice Research Datalink (CPRD) (GOLD and Aurum databases) for primary care records in the United Kingdom (UK), the Hospital Episode Statistics (HES) for secondary care inpatient records in England and the Office for National Statistics (ONS) in the UK. CPRD is considered representative to the UK population in terms of age, gender and ethnicity [7] and the ONS is considered the gold standard for delineating cause of death by suicide [8]. All participants in our study have data eligible for linkage with the three databases (CPRD, HES and ONS). Suicide cases (and open verdicts) were collated from the ONS, and all suicide cases (and open verdicts) identified during the study period were included in the analysis.

Controls were drawn from the sampling frame of CPRD subjects linkable to HES and ONS data. We matched controls to cases based on general practice and controls were randomly selected from the population of those who were at risk at the date of suicide death of the suicide case (risk-set sampling). For maximal statistical efficiency, every suicide case (14,515 in total) was matched with up to 40 live controls. We were able to match 40 controls for each of 14,240 (98.1%) suicide cases, and between 9 and 39 controls for each of the remaining 275 (1.9%) suicide cases. We only included patients who were at least 15 years old at the day of suicide or corresponding index date, have at least 1 year of records before the suicide index date, and have records deemed as acceptable quality for research. We also only selected patients from general practices classified as up to standard (for CPRD GOLD) in their quality of data recording.

### Outcome

Our outcome of interest was death due to suicide. These were identified from the ONS using the following International Classification of Diseases 10th revision (ICD-10) codes: X60-84, Y10-34 (excluding Y33.9), Y87.0, and Y87.2.

### Exposure

Our exposure of interest was diagnosis of HD. We used records from CPRD and HES for codes that confirm the diagnosis of HD including those referring to Huntington’s chorea and Huntington’s disease dementia. Please refer to Supplementary information for HD codes used in this study. Records of HD were collected from the earliest available in CPRD and HES up to the suicide/index date.

### Covariates

Covariates in our study include age (at suicide date for cases and at index date for controls) and gender.

### Statistical analysis

Odds ratios (ORs) were estimated using conditional logistic regression. Given that the study was risk-set sampled, ORs represent rate ratios [9]. The multivariable model was adjusted for gender and age at suicide or index date (fitted as a categorical variable) as a priori confounders. Further analysis was completed to examine how associations with suicide were modified by gender and age (using the median age of death from suicide in the HD cases as the cut-off point). To explore for interactions with gender and age, a likelihood ratio test was employed. STATA v.16 was employed for analysis. The study was approved by the MHRA Independent Scientific Advisory Committee (reference number 20_186RA).

## Results

Our sample included 594,674 patients, of which 14,515 (2.4%) were cases who died from suicide and 580,159 (97.6%) were controls (Table 1). There were 69 patients identified with a diagnosis of HD; of those 69 patients 12 patients (17.4%) were recorded as having died from suicide.

**Table 1:** Descriptive statistics for patients with Huntington’s disease compared to the whole sample

	Huntington’s disease (*N* = 69)		Whole sample (*N* = 594,675)	
	Suicide cases	Control	Suicide cases	Control
All n (%)	12 (17.4%)	57 (82.6%)	14,515 (2.4%)	580,159 (97.6%)
Median age at death in years^a^ (IQR)	45.8 (39.9–53.2)	72.2b (59.5–78.3)	47.4 (36.0–59.7)	81.55^c^ (71.9–88.4)

*IQR* interquartile range

^a^Mortality data for controls were obtained from the Clinical Practice Research Datalink. These controls were alive at the date of matching with cases but have died due to other causes thereafter

^b^20 out of 57 (35%) patients with Huntington’s disease have causes of death other than suicide

^c^49,920 out of 580,159 (8.6%) controls have causes of death other than suicide

The median age of death for those who died from suicide amongst patients with HD was 45.8 years (interquartile range IQR 39.9–53.2). This was significantly lower (*p* < 0.001) than those with HD who died from other causes whose median age at death was 72.3 years (IQR 59.5–78.3). Moreover, the median age of death for patients who died from suicide with HD (45.8 years, IQR 39.9–53.2) was significantly lower (*p* < 0.001) than the median age of death for patients who died from suicide in the whole sample without a diagnosis of HD (47.4 years, IQR 36.0–59.7).

As presented in Table 2, patients with HD were 9.2 (95% CI 4.9–17.4) times more likely to die from suicide compared with those who did not have HD, after adjustment for age and gender. Age at suicide death significantly modified the risk of death from suicide in individuals with a diagnosis of HD (*p* < 0.05). The higher suicide risk was highest in the younger age groups who were 45.8 years or younger at suicide/index date (OR 54.5, 95% CI 10.8–276.1) compared to those without HD diagnosis. There was no evidence of a significant interaction between gender and a diagnosis of HD in determining the risk of death by suicide (*p* > 0.1).

**Table 2:** Odds ratio for suicide in patients with Huntington’s disease compared to individuals without Huntington’s disease

	Huntington’s disease OR (95% CI)	*P* value*
All^a^	9.2 (4.9–17.4)	–
Gender		
Males	11.89 (5.2–26.9)	
Females	6.75 (1.8–25.1)	0.6
Age at suicide death		
≤ 45.8 years	54.53 (10.8–276.1)	
> 45.8 years	5.59 (2.3–13.7)	0.003

*p values based on the likelihood ratio test for testing for interaction

^a^Multivariable model adjusted for gender and age

## Discussion

Risk of death from suicide was markedly elevated in individuals with HD compared to individuals without this diagnosis. This risk was higher in younger individuals.

This study is the first to date to quantify the effect of HD on suicide risk compared to the general population using a large population-based dataset. The data quality for suicide records (using the gold standard ONS to identify cases) and for HD are additional strengths in the study. Primary care records in the UK are updated using information and diagnoses sent by secondary care, and are the main data repository for each individual patient. We would anticipate that a diagnosis such as HD would be correctly coded and recorded as such, but accept that some patients may be omitted, especially if they were seen outside the UK public health system for personal reasons.

Despite these strengths, we acknowledge that there are several limitations in the study. Using large population-based datasets allows hypotheses to be explored with sufficient numbers to observe associations that may not be detectable in smaller numbers of patients from the clinical context. However, there are few data on clinical status or disease severity in these larger datasets which does mean that there is little information as to the phenotypic presentation of HD and hence our analysis is likely to cover a spectrum of patients from HD gene carriers to those with severe clinical manifestations of HD. Using a combination of primary care (CPRD), secondary care (HES) and national mortality data (ONS) means that these data are probably the best available population-based data outside clinical cohorts, which will tend to be smaller and hence have less power to observe associations. The high odds ratios along with the consistency of these observations with clinical experience suggest that these are causal effects, and not a consequence of residual confounding.

The findings of HD are consistent with previous studies that suggested an association between HD and increased suicide risk [2–4, 6]. Excessive suicide risk in HD could be mediated by psychiatric disturbances, a core manifestation in HD and a robust risk factor for suicide [10].

As an autosomal dominant disease, patients with HD have often witnessed the suffering of a family member throughout the course of the disease and, in the absence of current cure, patients discern that this is their fate too. With a 50% probability of passing down the gene to offspring, patients may be distressed about the future of their children should they inherit the gene. These can all be additional contributing factors for increased suicide risk.

The particularly elevated risk in patients dying from suicide at a relatively younger age may be in part a reflection of disease severity and/or the bleak natural history of HD. Our data did not have reliable timing of the onset of either the clinical manifestations or the rate of progression of HD, but we speculate that an earlier age of HD onset has been associated with a faster deterioration of the disease [11], and hence those patients who died from suicide at a younger age may have been a subgroup enduring a more severe form of HD.

In summary, this is the first epidemiological study to demonstrate the size of effect of HD at the population-level on the risk of suicide. Routine assessment of suicide risk amongst patients diagnosed with HD is advisable, in both primary and secondary care settings, especially in younger patients. Given that more than three-quarters of patients with HD did not die from suicide, further research is required to understand, amongst those individuals, what constitutes potential protective factors from suicide.

## Supplementary Information

Below is the link to the electronic supplementary material.

Supplementary file1 (DOCX 16 kb)

## Data Availability

The data used for this work were obtained under license from CPRD. This license does not permit further sharing. However, anyone wishing to access the data can obtain it direct from CPRD subject to their licensing requirements.

## References

[1] Rodrigues FB, Abreu D, Damásio J, Goncalves N, Correia-Guedes L, Coelho M (2017). Survival, mortality, causes and places of death in a European Huntington’s disease prospective cohort. Mov Disord Clin Pract.

[2] Di Maio L, Squitieri F, Napolitano G, Campanella G, Trofatter JA, Conneally PM (1993). Suicide risk in Huntington’s disease. J Med Genet.

[3] Farrer LA, Opitz JM, Reynolds JF (1986). Suicide and attempted suicide in Huntington disease: Implications for preclinical testing of persons at risk. Am J Med Genet.

[4] Schoenfeld M, Myers RH, Cupples LA, Berkman B, Sax DS, Clark E (1984). Increased rate of suicide among patients with Huntington’s disease. J Neurol Neurosurg Psychiatry.

[5] World Health Organization (2019). Suicide in the world: global health estimates.

[6] Erlangsen A, Stenager E, Conwell Y, Andersen PK, Hawton K, Benros ME (2020). Association between neurological disorders and death by suicide in Denmark. JAMA.

[7] Herrett E, Gallagher AM, Bhaskaran K, Forbes H, Mathur R, van Staa T (2015). Data resource profile: clinical practice research datalink (CPRD). Int J Epidemiol.

[8] Thomas KH, Davies N, Metcalfe C, Windmeijer F, Martin RM, Gunnell D (2013). Validation of suicide and self-harm records in the Clinical Practice Research Datalink. Br J Clin Pharmacol.

[9] Rothman KJ, Greenland S, Lash TL (2008). Modern epidemiology.

[10] Franklin JC, Ribeiro JD, Fox KR, Bentley KH, Kleiman EM, Huang X (2017). Risk factors for suicidal thoughts and behaviors: a meta-analysis of 50 years of research. Psychol Bull.

[11] Foroud T, Gray J, Ivashina J, Conneally PM (1999). Differences in duration of Huntington’s disease based on age at onset. J Neurol Neurosurg Psychiatry.

