# Supplementary material for: Risk of mortality from suicide in patients with Huntington’s disease is increased compared to the general population in England
Source: J Neurol. 2022 Mar 27;269(8):4436–9. doi: 10.1007/s00415-022-11085-z (PMC9293836; doi:10.1007/s00415-022-11085-z)
Supplement: Supplementary file 1 — Supplementary file1 (DOCX 16 kb) [file 415_2022_11085_MOESM1_ESM.docx]

## Supplementary information.

**Codes for the outcome and exposure variables.**

**Codes used for the outcome variable suicide death**

Suicide cases were identified from the Office for National Mortality using the following International Classification of Diseases 10^th^ revision (ICD-10) codes: X60-84, Y10-34 (excluding Y33.9), Y87.0, and Y87.2.

**Codes used for the exposure variables**

Records of patients with Huntington’s disease were identified using codes from Clinical Practice Research Datalink (GOLD and AURUM) and ICD-10 codes in Hospital Episode Statistics.

Read codes used for Huntington’s disease (for CPRD GOLD):

- F134.00
- Eu02200

Medical code IDs used for Huntington’s disease (for CPRD Aurum)

- 97642017
- 295687017
- 7263011000006112
- 7263021000006116
- 3454551000006117
- 3454541000006119
- 3454531000006112

ICD-10 code used for Huntington’s disease (for Hospital Episode Statistics):

- G10
- F02.2
